# Comparison of Accommodative Response After Reading from Electronic Devices versus Hardcopy

**DOI:** 10.22599/bioj.483

**Published:** 2026-04-09

**Authors:** Ruchika Sah, Manish Sah, Gayathri Prabhu Rajiva

**Affiliations:** 1 Ramlal Golchha Eye Hospital Foundation, Nepal; 2 Laservision Refractive Surgery Center, Nepal; 3 Bubblevision Care, India

**Keywords:** amplitude of accommodation, hardcopy, electronic devices, accommodative facility

## Abstract

**Aim:**

To assess the change in accommodative response between electronic devices and hardcopy text after prolonged reading.

**Method:**

There were 30 participants (N = 30), with a mean age of 24.29 ± 5.21 years. The accommodative response (lag or lead of accommodation, amplitude of accommodation, and accommodative facility) was measured before and after reading for 60 minutes from a printed book, a smartphone, and a laptop, with a 24-hour break between tasks. Secondary outcomes included comparisons of accommodative responses between hardcopy text and smartphone, smartphone and laptop, and laptop and hardcopy text.

**Results:**

Accommodative lag was initially 0.50 ± 0.00 D, with significant increases observed after 60 minutes of reading from a smartphone (1.25 ± 0.50 D), a laptop (1.75 ± 0.50 D), and a hardcopy text (1.50 ± 0.25 D). A greater lag was noted with laptop use compared to smartphone reading (P < 0.01). Accommodative facility was significantly reduced when reading from a laptop compared to both a smartphone (P < 0.01) and a hardcopy (P < 0.01). The binocular mean amplitude of accommodation was –7.50 ± 1.25 D with the laptop and –8.00 ± 1.00 D with the hardcopy, with a P-value of 0.085, indicating no statistically significant difference.

**Conclusion:**

Prolonged near work significantly affects accommodative function, with laptops inducing the greatest accommodative lag and reduction in facility. Hardcopy reading preserved accommodative facility better than digital devices, while amplitude of accommodation showed minimal change. These results suggest that sustained laptop use may lead to greater visual strain compared to smartphones or printed text.

## Introduction

The usage of digital devices has become an integral element of daily life. Many individuals utilize these devices in practically all aspects of their professional and personal lives. The number of people using digital devices grows year after year. A total of 5.44 billion people use the internet globally, making up 67.1 percent of the total population (Petrosyan, 2024). People of all ages increasingly utilize digital devices for social and professional purposes, frequently for several hours every day. Nowadays, the design of digital electronic devices is not limited to desktop computers but also includes laptops, tablets, and smartphones, which can operate in any location. Related research indicates that up to 90% of individuals who work extensively with computers experience both visual and non-visual symptoms (Rosenfield, 2011). These symptoms may arise from non-ocular factors, such as improper equipment setup, or ocular factors, including uncorrected refractive errors, adaptation to computer screens, or convergence fatigue.

Numerous investigations have sought to determine the association between digital display usage and clinically observable reductions in visual performance (Portello *et al.*, 2009; Shrestha *et al.*, 2011). Shrestha *et al.* (2011) identified accommodative infacility as the most common visual dysfunction in video display terminal (VDT) users, who reported an average daily computer use of nearly seven hours. This finding underscores the potential impact of prolonged screen exposure on accommodative function. Benedetto *et al.* (2013) conducted a study with twelve subjects who engaged in 70-minute reading sessions using LCD, e-ink, and hardcopy devices at a viewing distance of 60 cm. The study revealed a significantly lower mean blink rate during use of the LCD device compared to both the e-ink and hardcopy devices.

Lag of accommodation is directly proportional to the near viewing period. Greater lag was found with iPads compared to printed text in Hue *et al.*’s investigation (2014). Varying working distances and job specifics, such as text size and contrast, may place distinct pressures on the accommodation and vergence systems, impacting the accommodative facility differentially. Smartphones and tablets are frequently used simultaneously with other activities. To maintain good vision, the user must quickly shift accommodation to focus on the screen and then relax accommodation for distant objects. Golebiowski *et al.* (2020) reported that after using a smartphone for 60 minutes, binocular accommodative facility reduced considerably from 11.3 (IQR 6.6) cycles/min pre-task to 7.8 (2.5) cycles/min post-task (P = 0.01). With the rising usage of these and comparable devices for prolonged close work, it is unknown whether the concerns typically mentioned with viewing desktop and laptop displays are as widespread with these devices. Similarly, several studies have compared smartphones with various other electronic devices for shorter durations.

When comparing before and after near-vision activities on an iPad screen against a laptop, Phamonvaechavan *et al.* (2017) found substantial decreases in accommodative amplitude. After performance of near-vision tasks, Cisarik and Nguyen’s investigation (2019), using an open-field autorefractor, revealed no discernible changes in accommodative response (MEM) between the iPad and the laptop. According to Park *et al.* (2014), the accommodating lag after smartphone use was substantially greater than after book reading, and the monocular and binocular accommodative amplitude was much lower after smartphone use than after reading. In recent years, the use of smartphones for different tasks has increased significantly. With rapid technological advancements, people’s preferred ways of reading have also changed—from printed books to e-books. Different activities place different demands on our eyes, so it is important to understand how visual functions, like accommodation, vary with different reading formats. This study aims to find out how accommodative response changes after reading for a long time on electronic devices compared to reading printed text.

## Methods

For the study, we included optometry students, interns, and some bachelor students who had come to the hospital for an eye checkup. Inclusion criteria for the study were that the individuals were aged 20–35 years, with best-corrected visual acuity of 6/6 and N6 monocular, screen time not exceeding eight hours per day, no history of ocular trauma or previous ocular surgeries, and were voluntarily willing to participate in the study. Exclusion criteria included any ocular pathology such as chronic infection or inflammation; a history of non-strabismic binocular vision problems or computer vision syndrome (CVS); vision therapy for these issues; and individuals with amblyopia, strabismus, or nystagmus. Before the study commenced, each patient provided informed consent, and the research was approved by the Institutional Review Committee of Tilganga Institute of Ophthalmology (04/2025) and was conducted in accordance with the Declaration of Helsinki. Each participant received a complete optometric test, which comprised an initial screening process and a detailed binocular vision evaluation. The initial screening protocol comprised history taking (ocular and systemic), Hirschberg’s test, extraocular motility assessment, cover test, and vision screening. After the initial examination had been completed, a binocular vision assessment was performed. Those with any binocular anomalies, i.e., non-strabismic binocular vision anomalies or any kind of strabismus, were excluded from the study and referred for further management, while those with normal binocular vision were included in the study.

Participants wearing spectacles were those who had refractive errors, while those without any kind of refractive error participated without spectacles in the study. They were asked to sit and read continuously for one hour using a smartphone, laptop, or hardcopy book. The room’s illumination was maintained at an average of 350 lux, consistent with recommended lighting levels for reading tasks and computer-based work. Illumination levels were verified and calibrated using a lux meter. Appropriate tables and seating were provided to participants to facilitate sustained reading from books and laptops, thereby ensuring ergonomic posture over a one-hour period. Similarly, provisions were made for reading from mobile devices, enabling participants to reduce musculoskeletal strain by resting their hands on the table throughout the session. All reading activities were systematically supervised by qualified optometrists for the entire duration of the study to ensure adherence to the study protocol. Participants could choose their preferred reading material or a novel for the reading session. For a continuous 60 minutes, each subject was asked to read aloud from a laptop screen (Dell laptop with a 15.6-inch HD, 1366 × 768, 60 Hz flat panel LED screen), a smartphone, or a hardcopy text at a viewing distance of 40 cm. The text was typed in a 12-point black Times New Roman font with a contrast of about 80%. The research employed an iPhone 6, which was mounted in a holder. The smartphone was positioned in a holder at an angle of 20–25 degrees below eye level. The device featured a 4.7-inch LCD display with a default font size of 15 points, operated in portrait orientation, with a resolution of 1334 × 750 pixels and a pixel density of 326 dots per inch (dpi). The printed manual was 6.0 inches, was produced on white paper, and had a font size of 10–12 points. All sessions utilized identical passages that were matched in size and contrast. The screen reading activities and the hardcopy text were each given 60 minutes, with a 24-hour rest in between. The conditions were counterbalanced (with alternating participants undertaking the laptop, smartphone, or hardcopy condition first), and each subject had three sessions that were at least 24 hours apart. All tasks were performed in the same room under consistent lighting conditions recommended for reading. Subjects were told to have a consistent viewing angle and working distance. After performing the 60-minute reading task for each condition (i.e., hardcopy text and electronic devices), accommodative responses, including accommodative amplitude, accommodative lag, and accommodative facility, were measured for each subject.

### Test procedures

The parameters measured in the study were the Monocular Estimation Method, amplitude of accommodation (AA), and accommodative facility, both uniocularly and binocularly. The Monocular Estimation Method of dynamic retinoscopy was performed under normal room illumination, and the patients were instructed to wear their habitual correction. They were instructed to keep the targets clear. The participant fixated on the reading device monocularly, positioned in the same plane as the retinoscope, which was placed at a distance of 40 cm and displayed letters of 20/30 size. The retinoscope beam was oriented vertically. While the patient was observing, the examiner swiftly moved the streak over the patient’s left and right eyes, attempting to assess the response for ‘with motion’ or ‘against motion’, or neutral. Neutralization of motion responses was achieved by applying a plus lens for the ‘with-motion’ condition and a minus lens for the ‘against-motion’ condition. The lens power that first made the reflex look neutral when quickly placed in front of the eye was noted. The difference in dioptres between the testing distance and this lens power was taken as the accommodative response. The negative lens method was used to quantify binocular and monocular amplitude of accommodation. In this procedure, negative lenses are added to the distance refractive correction until the participant loses clarity. The AA is calculated by adding the highest negative lens power and using a working distance lens of 2.50 D for 40 cm, using a target of 20/30 size. Both binocular and monocular accommodative facilities were assessed using a flipper of ±2.00 DS. The RAF rule set the target one line above the best-corrected visual acuity. The test was performed at a distance of 40 cm. We put the +2.00 DS lens in front of the subject’s eye and asked them to try to read the letters clearly. When the letters were deemed to be clear, the subject was directed to swiftly flip the flipper to the negative side. The participant was then asked to reread the letters and indicate when they were clear. We measured the number of cycles per minute for both monocular and binocular tests.

### Post-task evaluation

The post-task evaluations involved measuring all the accommodative parameters that were assessed in the pre-task evaluation, after 60 minutes of prolonged reading during all three sessions. Evaluations were conducted immediately upon completion of the reading task, without any rest period.

### Statistical analysis

Statistical analysis was performed using SPSS version 20. The Shapiro–Wilk test indicated that most outcome variables in this study did not follow a normal distribution. Therefore, a nonparametric Friedman test was applied to compare data within and across groups, with statistical significance set at P < 0.05.

## Results

The study was successfully completed by all 30 subjects, including 13 males and 17 females, with a mean age of 24.29 ± 5.21 years, and there were no dropouts. Statistically significant differences were found before and after reading from all the devices.

### Accommodative lag

The difference between the accommodative stimulus and the response was evaluated by measuring the accommodative lag using the Monocular Estimation Method. Accommodative lag was initially determined to be 0.50 ± 0.00 D, with significant changes observed after reading from a smartphone (1.25 ± 0.50 D), laptop (1.75 ± 0.50 D), and hardcopy text (1.50 ± 0.25 D) for 60 minutes (Table 1). A greater lag was observed when subjects read from the laptop (P < 0.01) compared to reading from the smartphone. Similarly, a greater lag was found when reading from the laptop compared with hardcopy text (P < 0.01). Furthermore, no significant change in accommodative lag was observed when comparing the smartphone with hardcopy text (P = 0.049) (Table 2).

**Table 1:** **Change in accommodative parameters at near, before and after viewing text**. MEM: Monocular Estimation Method; AA: amplitude of accommodation; ACC. Facility: accommodation facility; OD: oculus dexter; OS: oculus sinister; OU: oculus uterque; D: diopter; cpm: cycles per minute.


PARAMETERS	PRE (MEAN ± SD)	POST (MEAN ± SD)			P-VALUE		
	
		SMARTPHONE	LAPTOP	HARDCOPY	SMARTPHONE	LAPTOP	HARDCOPY

MEM OD (D)	0.50 ± 0.00	1.25 ± 0.50	1.75 ± 0.50	1.50 ± 0.25	P < 0.005	P < 0.005	P < 0.005

OS (D)	0.50 ± 0.25	1.25 ± 0.50	1.75 ± 0.50	1.50 ± 0.50	P < 0.005	P < 0.005	P < 0.005

AA OD (D)	–8.00 ± 1.00	–7.50 ± 1.00	–7.50 ± 1.00	–8.0 ± 1.50	P < 0.005	0.009	0.490

OS (D)	–8.00 ± 1.50	–7.50 ± 1.00	–7.50 ± 1.00	–8.0 ± 1.00	P < 0.005	0.006	0.480

OU (D)	–8.50 ± 1.00	–8.00 ± 1.00	–7.50 ± 1.25	–8.00 ± 1.00	0.026	0.036	0.388

ACC. FACILITY OD (cpm)	12.0 ± 3.00	10.00 ± 2.00	9.00 ± 1.00	10.00 ± 2.00	P < 0.005	P < 0.005	P < 0.005

OS (cpm)	13.00 ± 2.00	10.00 ± 2.00	9.50 ± 1.50	10.00 ± 2.00	P < 0.005	P < 0.005	P < 0.005

OU (cpm)	13.00 ± 2.00	11.00 ± 2.00	10.00 ±1.00	11.00 ± 2.50	P < 0.005	P < 0.005	P < 0.005


**Table 2:** **Comparison of accommodative parameters of smartphone with laptop and hardcopy**. MEM: Monocular Estimation Method; AA: amplitude of accommodation; ACC. Facility: accommodation facility; OD: oculus dexter; OS: oculus sinister; OU: oculus uterque; D: diopter; cpm: cycles per minute.


PARAMETERS	SMARTPHONE (MEAN ± SD)	POST (MEAN ± SD)		P-VALUE	
	
		LAPTOP	HARDCOPY	LAPTOP	HARDCOPY

MEM OD (D)	1.25 ± 0.50	1.75 ± 0.50	1.50 ± 0.25	P < 0.005	0.049

OS (D)	1.25 ± 0.50	1.75 ± 0.50	1.50 ± 0.50	P < 0.005	0.039

AA OD (D)	–7.50 ± 1.00	–7.50 ± 1.00	–8.0 ± 1.50	0.161	0.016

OS (D)	–7.50 ± 1.00	–7.50 ± 1.00	–8.0 ± 1.00	0.043	P < 0.005

OU (D)	–8.00 ± 1.00	–7.50 ± 1.25	–8.0 ± 1.00	0.791	0.146

ACC. FACILITY OD (cpm)	10.00 ± 2.0	9.00 ± 1.00	10.00 ± 2.00	P < 0.005	0.965

OS (cpm)	10.00 ± 2.00	9.50 ± 1.50	10.00 ± 2.50	P < 0.005	0.015

OU (cpm)	11.00 ± 2.00	10.00 ± 1.00	10.00 ± 2.50	P < 0.005	0.015


### Amplitude of accommodation

Monocular amplitude of accommodation significantly changed after 60 minutes of reading from both a smartphone and a laptop (Table 1). For both eyes (OU), the mean AA was –7.50 ± 1.25 dioptres with a laptop and –8.00 ± 1.00 dioptres with a hardcopy, with a P-value of 0.085, which is not statistically significant (Table 3).

**Table 3:** **Comparison of accommodative parameters in laptop vs. hardcopy**. MEM: Monocular Estimation Method; AA: amplitude of accommodation; ACC. Facility: accommodation facility; OD: oculus dexter; OS: oculus sinister; OU: oculus uterque; D: diopter; cpm: cycles per minute.


PARAMETERS	LAPTOP (MEAN ± SD)	HARDCOPY (MEAN ± SD)	P-VALUE

MEM OD (D)	1.75 ± 0.50	1.50 ± 0.50	P < 0.005

OS (D)	1.75 ± 0.50	1.50 ± 0.50	P < 0.005

AA OD (D)	–7.50 ± 1.00	–8.0 ± 1.00	0.022

OS (D)	–7.50 ± 1.00	–8.0 ± 1.00	0.022

OU (D)	–7.50 ± 1.25	–8.00 ± 1.00	0.085

ACC. FACILITY OD (cpm)	9.00 ± 1.00	10.00 ± 2.00	P < 0.005

OS (cpm)	9.50 ± 1.50	10.00 ± 2.00	P < 0.005

OU (cpm)	10.00 ± 1.00	11.00 ± 2.50	P < 0.005


### Accommodative facility

The accommodative facility was examined in both monocular and binocular vision. Monocularly, the accommodative facility was initially measured at 12.0 ± 3.0 cycles per minute. A significant reduction was observed after reading from a smartphone (10.0 ± 2.0), laptop (9.0 ± 1.0), and hardcopy text (10.0 ± 2.0) for 60 minutes (Table 1). Accommodative facility was significantly reduced when reading from a laptop compared to a smartphone (P < 0.01) and reduced when reading from a laptop compared to a hardcopy (P < 0.01) (Table 3). However, no significant changes were found when comparing the smartphone to the hardcopy; the accommodative facility was similar for both. The study found that accommodation facility was significantly higher when using a hardcopy compared to a laptop (Table 3). The accommodation facility data show significant differences between reading from a laptop and a hardcopy. For the right eye (OD), the mean was 9.00 ± 1.00 cycles per minute with a laptop and 10.00 ± 2.00 cycles per minute with a hardcopy (P < 0.005).

## Discussion

In recent years, a huge variety of portable electronic devices has entered the market, with tablets and smartphones, as well as personal computers, being the most widely used. Several studies have shown that such electronic devices are becoming increasingly popular in people’s daily lives.

Moulakaki *et al.* (2017) reported no significant differences in accommodative responses after 10 minutes of reading from a smartphone and tablet. Similarly, Hue *et al.* measured the accommodative parameter with an infrared optometer and found no significant change in response over the 12-minute reading time for any of the conditions tested (Hue *et al.*, 2014). This may be due to the effect of shorter task durations (Cisarik and Nguyen, 2019). Tosha *et al.* reported that accommodative lag increased in subjects who experienced greater visual discomfort, particularly during extended fixation of at least 30 seconds. Thus, viewing a smartphone or laptop at a closer distance or on a smaller screen for a prolonged period, such as 60 minutes, may lead to a larger accommodative lag (Park *et al.*, 2014; Tosha *et al.*, 2009). The present study found a significantly greater lag of accommodation after reading from a smartphone, laptop, or hardcopy for a 60-minute period. In the present study, a higher accommodative lag was observed while reading printed text. This finding may be attributed to prolonged viewing of small font sizes without adequate intermittent breaks, which can increase the demand on the accommodative system, disrupt its dynamic function, and result in a greater lag of accommodation (Padavettan *et al.*, 2021). The increased accommodative lag may also reflect the visual fatigue associated with sustained near work involving smartphones and laptop use (Chellapan *et al.*, 2024). In addition, the high luminance of digital displays and light reflections from screens may further strain the accommodative mechanism, thereby contributing to reduced accommodative efficiency (Ha *et al.*, 2014). Wick and Morse (2002) reported an increased lag of accommodation when viewing a laptop monitor compared to a smartphone, which is similar to our study. Hynes *et al.* (2022) found that displays with higher pixel density, such as smartphones compared to laptops, may help reduce the lag in accommodation. Liang *et al.* (2021) reported no significant change in accommodative lag when comparing reading from a smartphone and hardcopy, which is consistent with our study.

Prolonged use of VDT is known to cause visual discomfort in workers, manifesting as eye fatigue, irritation, redness, blurred vision, and diplopia during or after work. Yoo *et al.* reported that 90 minutes of computer use in Korean adults led to reduced blinking, resulting in an increased near point accommodation, decreased amplitude of accommodation, and elevated accommodative stress immediately after the task (Yoo *et al.*, 1992). Accommodative amplitude at near vision after reading text on a smartphone or desktop computer significantly decreased compared to that before reading in the current study. This is similar to the study by Phamonvaechavan *et al.* (2017), which reveals that the amplitude of accommodation dramatically lowers when reading from an iPad or laptop. The current study also demonstrates that reading from a smartphone lowers the amplitude of accommodation substantially more than reading from hardcopy material. This conclusion is congruent with that of Park *et al.* (2014), who found that the amplitude of accommodation was considerably reduced immediately after using a smartphone compared to reading from hardcopy material. The reduction in amplitude of accommodation observed in this study may be attributed to impairment of tonic accommodation resulting from prolonged near work (Padavettan *et al.*, 2021; Kang *et al.*, 2021). Narawi *et al.* (2020) reported that 20 minutes of smartphone use caused a significant decrease in amplitude of accommodation, which was associated with increased accommodative lag among participants. Our study also found a higher accommodative lag following smartphone and laptop use. Viewing smartphones at closer working distances may place continuous strain on the crystalline lens and ciliary muscles, leading to accommodative adaptation and visual fatigue (Chellapan *et al.*, 2024). However, significant changes in the amplitude of accommodation were found only for the left eye when comparing the smartphone with hardcopy. The right eye showed no significant changes. This might be due to ocular fatigue caused by sustained near viewing, which could have biased the findings. Similarly, the present study shows a significant decrease in the amplitude of accommodation monocularly with both smartphones and laptops. Binocularly, however, no significant changes were observed, possibly due to the effect of vergence. Further studies should focus on vergence parameters. Yoo *et al.* reported less blinking during 90 minutes of laptop work, reduced amplitude of accommodation, and an extended period of accommodative tension in Korean adults immediately after laptop use, which is similar to the present study (Yoo *et al.*, 1992).

Accommodative facility is a typical clinical test that measures changes in accommodation to different stimuli near and at distance. Reading via a smartphone, laptop, or hardcopy reduced both monocular and binocular accommodative facility significantly. This finding aligns with various studies that have reported a statistically significant reduction in binocular accommodative facility after 60 minutes of smartphone reading (Golebiowski *et al.*, 2020; Kim *et al.*, 2017a). According to Golebiowski *et al.* (2020), the decline in accommodative facility parameters may be strongly influenced by reduced vergence facility. A significant reduction in accommodative facility was observed among participants with convergence insufficiency following smartphone use. This reduction may be attributed to inadequate convergence, resulting in delayed relaxation and increased simultaneous demands on both convergence and accommodation to maintain clear vision (Kim *et al.*, 2017b). Similarly, Kaliugavaradhan and Ramamurthy (2023) reported a significant 6% reduction in monocular accommodative facility after a one-hour near task, with the negative response time (time taken to clear plus lenses) delayed by approximately 77%. These findings indicate difficulty in relaxing accommodation, likely due to a near work-induced transient effect. In our study, binocular accommodative facility decreased by approximately two cycles after smartphone use and three cycles after laptop use. These findings are consistent with previous research. Padavettan *et al.* (2021) reported a two-cycle reduction after 30 minutes of smartphone reading, while Golebiowski *et al.* (2020) observed a 3.5-cycle decrease after one hour of smartphone use. Similarly, Park *et al.* (2014) found a one-cycle reduction in monocular accommodative facility after one hour of computer reading. Our results support earlier studies indicating that one hour of electronic device use significantly reduces accommodative facility, regardless of the device type. Rosenfield *et al.* (2010) assessed monocular and binocular accommodative facility using ±2.00 D flippers before and after a 25-minute laptop task at a viewing distance of 50 cm. After the activity, there was no significant change in either monocular or binocular accommodative ability, which might be attributable to the shorter length of reading time (Rosenfield *et al.*, 2010).

Another finding of this study is that both monocular and binocular accommodative facility slightly decreased when reading from a laptop compared with a smartphone and hardcopy. This is because reading from a laptop or hardcopy for a sustained period of 60 minutes increased the ability to relax accommodation more than the effect of smartphone use in the same working environment. A similar decrease in accommodative facility after two hours of laptop work was reported by Saito *et al.* (1994). Kim *et al.* (2017a), who also investigated reading text, found reduced binocular accommodative facility. Vergence ability has a considerable impact on binocular accommodative facility outcomes; therefore, any changes seen might be the consequence of changes in vergence facility or vergence ranges. More research on the impact of smartphone use on accommodative facility should incorporate vergence tests in addition to monocular and binocular accommodative facility assessments. Monocular accommodative facility testing would allow for the examination of focus adjustment abilities while avoiding the impacts of vergence skills.

### Clinical implications

The findings of this study suggest that reading from electronic devices imposes a greater accommodative demand than reading hardcopy text. However, implementing a recommendation to preferentially use printed materials may be challenging in the contemporary digital era, where electronic devices are deeply embedded in education, professional activities, and daily life. Instead of advocating complete avoidance of electronic text, clinical emphasis should be placed on patient education regarding healthy digital viewing practices. These include maintaining an appropriate working distance, using adequate font sizes, taking regular visual breaks, and optimizing screen ergonomics. From a public health perspective, strategies should prioritize awareness initiatives that promote safe digital viewing behaviours, such as limiting continuous screen time, adopting ergonomic adjustments, and ensuring periodic visual rest. Incorporating eye health education into school curricula and workplace wellness programmes may contribute to reducing the prevalence of digital eye strain and accommodative dysfunction, thereby enhancing visual comfort and overall productivity.

### Limitations

There are some limitations to our current study. There were some uncontrolled factors influencing accommodation, such as the position or angle of the smartphone and the reading position of the subjects while viewing the text. The visual angle while reading from a book, a laptop, or a smartphone was not fixed. Vergence parameters and symptoms were not assessed. We did not complete the Visual Fatigue Symptom Survey or measure the blink rate before and after the reading task. Including these measurements could have provided deeper insight and a more comprehensive explanation of our findings.

### Future research

Future research should examine the effects of prolonged electronic device use on accommodation and vergence in diverse populations, including children, older adults, and working professionals. Studies in real-world settings incorporating measures such as blink rate, ocular surface health, and dynamic accommodation, as well as evaluating ergonomic interventions and visual breaks, could provide practical strategies to reduce accommodative stress and guide safe device use across different age groups and occupations.

## Conclusion

Prolonged use of near devices such as smartphones, laptops, and hardcopy materials induces the following changes in accommodative function: (1) reduced accommodative amplitude, (2) increased accommodative lag, and (3) reduced accommodative facility. Further studies on visual functions during binocular viewing, such as positive/negative fusional vergence, fusional vergence amplitude, relative vergence, and vertical phoria, are necessary to fully understand the correlation between subjective/objective symptoms and the effects on visual function caused by extended use of various near devices. The most significant changes in accommodative function were observed after laptop use, followed by smartphone use and hardcopy reading at a similar workstation. This study suggests that reading from hardcopy materials should be promoted over electronic devices, as the latter can adversely affect accommodative function.

## References

[1] Benedetto, S., Drai-Zerbib, V., Pedrotti, M., Tissier, G. and Baccino, T. (2013) ‘E-readers and visual fatigue’, PloS one, 8, e83676. (Accessed: DD Month YYYY). Available at: 10.1371/journal.pone.0083676 24386252 PMC3873942

[2] Chellapan, T., Daud, N.M. and Narayanasamy, S. (2024) ‘Smartphone use patterns and the impact on accommodation and convergence system of the eyes among Malaysian teenagers’, International Journal of Ophthalmology, 17(11), 2093. Available at: 10.18240/ijo.2024.11.16 39559302 PMC11528288

[3] Cisarik, P.M. and Nguyen, J. (2019) ‘Comparison of Accommodative Responses to e-Ink vs. LCD vs. Standard Ink on Hard Copy’, Optometry & Visual Performance, 7.

[4] Golebiowski, B., Long, J., Harrison, K., Lee, A., Chidi-Egboka, N. and Asper, L. (2020) ‘Smartphone use and effects on tear film, blinking and binocular vision’, Current Eye Research, 45, 428–434. Available at: 10.1080/02713683.2019.1663542 31573824

[5] Ha, N.-R., Kim, C.-J., Jung, S.A., Choi, E.J. and Kim, H.J. (2014) ‘Comparison of accommodative system according to the material and font size of near visual media’, Journal of Korean Ophthalmic Optics Society, 19, 217–224. Available at: 10.14479/jkoos.2014.19.2.217

[6] Hue, J.E., Rosenfield, M. and Saá, G. (2014) ‘Reading from electronic devices versus hardcopy text’, Work, 47, 303–307. Available at: 10.3233/WOR-131777 24284668

[7] Hynes, N.J., Cufflin, M.P., Hampson, K.M. and Mallen, E.A. (2022) ‘The effect of image resolution of display types on accommodative microfluctuations’, Ophthalmic and Physiological Optics, 42, 514–525. Available at: 10.1111/opo.12949 35107178 PMC9302673

[8] Kaliugavaradhan, A. and Ramamurthy, D. (2023) ‘Accommodative facility and response time before and after computer task of varying durations in young adults’, The British and Irish Orthoptic Journal, 19, 85. Available at: 10.22599/bioj.295 37868656 PMC10588489

[9] Kang, J.W., Chun, Y.S. and Moon, N.J. (2021), ‘A comparison of accommodation and ocular discomfort change according to display size of smart devices’, BMC ophthalmology, 21, 44. Available at: 10.1186/s12886-020-01789-z 33461518 PMC7814593

[10] Kim, J., Um, J.Y., Sung, H.N., Kim, S.R. and Park, M. (2017a) ‘Changes in accommodative function after reading with paper book and e-book on tablet PC’. J Korean Ophthalmic Opt Soc, 22, 183–190. Available at: 10.14479/jkoos.2017.22.2.183

[11] Kim, S.R., Kwak, H., Kang, M.S., Kim, S.I. and Park, M. (2017b) ‘The changes in convergence function of accommodative anomalies in their twenties after watching video on a smartphone’, J Korean Ophthalmic Opt Soc, 22, 133–142. Available at: 10.14479/jkoos.2017.22.2.133

[12] Liang, X., Wei, S., Li, S.-M., An, W., Du, J. and Wang, N. (2021) ‘Effect of reading with a mobile phone and text on accommodation in young adults’, Graefe’s Archive for Clinical and Experimental Ophthalmology, 259, 1281–1288. Available at: 10.1007/s00417-020-05054-3 PMC8102294 33464380

[13] Moulakaki, A.I., Recchioni, A., Águila-Carrasco, A.J.D., Esteve-Taboada, J.J. and Montés-Micó, R. (2017) ‘Assessing the accommodation response after near visual tasks using different handheld electronic devices’, Arquivos brasileiros de oftalmologia, 80, 9–13. Available at: 10.5935/0004-2749.20170004 28380093

[14] Narawi, W.S., Razak, S.A. and Azman, N. (2020) ‘The effect of smartphone usage on accommodation status’, Malaysian Journal of Medicine and Health Sciences, 16(2), 244–247.

[15] Padavettan, C., Nishanth, S., Madhivanan, N. and Madhivanan, N. (2021) ‘Changes in vergence and accommodation parameters after smartphone use in healthy adults’, Indian Journal of Ophthalmology, 69, 1487–1490. Available at: 10.4103/ijo.IJO_2956_20 34011725 PMC8302311

[16] Park, M., Ahn, Y.J., Kim, S.J., You, J., Park, K.E. and Kim, S.R. (2014) ‘Changes in accommodative function of young adults in their twenties following smartphone use’, Journal of Korean Ophthalmic Optics Society, 19, 253–260. Available at: 10.14479/jkoos.2014.19.2.253

[17] Petrosyan, A. (2024) Worldwide digital population 2024 [Online]. Statista. Available at: https://www.statista.com/statistics/617136/digital-population-worldwide/#:~:text=Worldwide%20digital%20population%202024&text=As%20of%20April%202024%2C%20there,percent%20of%20the%20global%20population (Accessed: 14 August 2024).

[18] Phamonvaechavan, P. (2017) ‘A comparison between effect of viewing text on computer screen and iPad® on visual symptoms and functions’, Siriraj Medical Journal, 69, 185–189.

[19] Portello, J., Chu, C., Rosenfield, M., Benzoni, J. and Collier, J. (2009) ‘Computer Vision Syndrome: Hard Copy versus Computer Viewing’, Investigative Ophthalmology & Visual Science, 50, 5333–5333.

[20] Rosenfield, M. (2011) ‘Computer vision syndrome: a review of ocular causes and potential treatments’, Ophthalmic and Physiological Optics, 31, 502–515. Available at: 10.1111/j.1475-1313.2011.00834.x 21480937

[21] Rosenfield, M., Gurevich, R., Wickware, E. and Lay, M. (2010) ‘Computer Vision Syndrome: Accommodative & Vergence Facility’, Journal of Behavioral Optometry, 21.

[22] Saito, S., Sotoyama, M., Saito, S. and Taptagaporn, S. (1994) ‘Physiological indices of visual fatigue due to VDT operation: Pupillary reflexes and accommodative responses’, Industrial health, 32, 57–66. Available at: 10.2486/indhealth.32.57 7806446

[23] Shrestha, G.S., Mohamed, F.N. and Shah, D.N. (2011) ‘Visual problems among video display terminal (VDT) users in Nepal’, Journal of Optometry, 4, 56–62. Available at: 10.1016/S1888-4296(11)70042-5

[24] Tosha, C., Borsting, E., Ridder, W.H. III. and Chase, C. (2009) ‘Accommodation response and visual discomfort’, Ophthalmic and Physiological Optics, 29, 625–633. Available at: 10.1111/j.1475-1313.2009.00687.x 19821926

[25] Wick, B. and Morse, S. (2002) ‘Accommodative accuracy to video display monitors: Poster# 28’, Optometry and Vision Science, 79, 218. Available at: 10.1097/00006324-200212001-0041311999147

[26] Yoo, J.S., Yoon, J.W. and Kim, J.D. (1992) ‘Influence of VDT work on accommodative function’, Journal of the Korean Ophthalmological Society, 693–397.

